# What are the Best Animal Models for Testing Early Intervention in Cerebral Palsy?

**DOI:** 10.3389/fneur.2014.00258

**Published:** 2014-12-04

**Authors:** Gavin John Clowry, Reem Basuodan, Felix Chan

**Affiliations:** ^1^Institute of Neuroscience, Newcastle University, Newcastle upon Tyne, UK

**Keywords:** cerebral palsy, corticospinal tract, hypoxia/ischemia, perinatal stroke, periventricular white matter injury

## Abstract

Interventions to treat cerebral palsy should be initiated as soon as possible in order to restore the nervous system to the correct developmental trajectory. One drawback to this approach is that interventions have to undergo exceptionally rigorous assessment for both safety and efficacy prior to use in infants. Part of this process should involve research using animals but how good are our animal models? Part of the problem is that cerebral palsy is an umbrella term that covers a number of conditions. There are also many causal pathways to cerebral palsy, such as periventricular white matter injury in premature babies, perinatal infarcts of the middle cerebral artery, or generalized anoxia at the time of birth, indeed multiple causes, including intra-uterine infection or a genetic predisposition to infarction, may need to interact to produce a clinically significant injury. In this review, we consider which animal models best reproduce certain aspects of the condition, and the extent to which the multifactorial nature of cerebral palsy has been modeled. The degree to which the corticospinal system of various animal models human corticospinal system function and development is also explored. Where attempts have already been made to test early intervention in animal models, the outcomes are evaluated in light of the suitability of the model.

## Introduction

It is widely accepted that research with animal models is crucial to developing and testing new therapies. We need to understand the cellular mechanisms that underlie the organism’s response to brain injury in the short and long term, and it is assumed that at the cellular level all mammals share these responses. However, there are drawbacks to this approach. It is important not to fall into the traps identified in pre-clinical adult stroke research, which may explain the massive failure rate in clinical trials of novel neuroprotective agents identified in animal experiments (1). These include omission of fundamental aspects of experimental design such as blinding, randomization, exclusion reporting, and sample size, but also “cherry picking” the data to publish to maximize impact (2). But it also seems to us that not enough time is spent asking how directly applicable to humans are our models?

Careful consideration has to be given as to the extent the animal model reflects human in terms of the way the nervous system functions and develops. Timing of experiments is crucial; for instance one of the significant drawbacks with studying rodents is the rapidity with which the CNS develops over days, compared to months in primate species, whereas, cellular processes of neuroinflammation are likely to occur on a more similar timescale between species. In this article, we ask what exactly are we trying to model? How similar are our animal models to the human condition? What have our animal models told us so far, and what outcomes should we be measuring in order to gage the likely success of our proposed therapies?

## What are We Trying to Model?

### Cerebral palsy in humans

**Figure 1:**
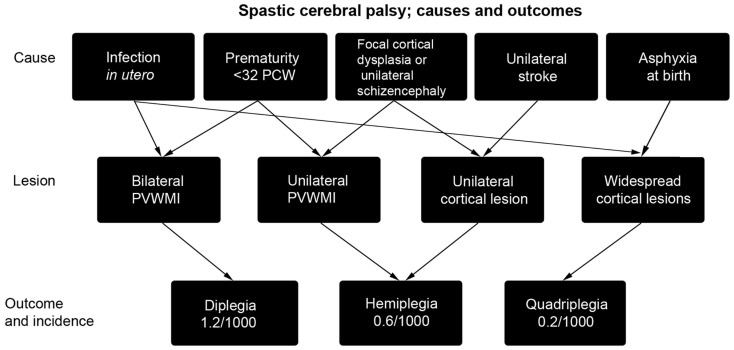
**A summary of the causes of spastic cerebral palsy, and the particular outcomes they lead to [reproduced with permission from Ref. (11)]**. Asphyxia at birth may arise from prolapsed cord, intrapartum hemorrhage, uterine rupture, or maternal cardiac arrest. As arrows indicate, multiple causes may combine to produce cerebral palsy (4) and may also interact with subtle genetic variations in individuals that cause predisposition to stroke (6). PCW, post-conceptional weeks; PVWMI, periventricular white matter injury.

The incidence of cerebral palsy in the developed world is high, around 2 per 1000 live births or more (3). It is therefore a common condition that causes disability throughout life, which is often severe. Cerebral palsy is an umbrella term for a number of conditions including cerebellar ataxia and basal ganglia disorders, but this article will largely concentrate on the most common condition, spastic cerebral palsy (80% of cases) primarily arising from insults to the cerebral cortex and associated, sub-cortical white matter (4). Causal pathways are many and may interact with each other, indeed multiple causes, including a genetic predisposition to infarction, may need to interact to produce a clinically significant injury (4–6). The most commonly encountered causes are summarized in Figure 1 and include periventricular white matter injury (PVWMI) in premature babies, which results from hypoxia/ischemia (H/I) in the periventricular regions around the lateral ventricles. This results, primarily, in damage to the subplate and developing sub-cortical axon tracts of the intermediate zone whilst the overlying gray matter is relatively spared. It generally causes spastic diplegia. In all, bilateral spasticity has a prevalence of 1.2/1000 live births (7). Unilateral spasticity and weakness is also common (prevalence 0.6/1000 live births) with roughly one-third of cases resulting from focal periventricular white matter lesions and one-third involving cortical or deep gray matter lesions, mainly as a result of infarcts of the middle cerebral artery. A further fifth of such cases result from brain maldevelopments, mainly focal cortical dysplasia or unilateral schizencephaly (8). More severe hypoxia or anoxia at the time of birth is associated with widespread injury of white and gray matter resulting in spastic quadraparesis along with severe cognitive deficits. In all cases, there is a progressive evolution of the movement disorder over months and years. Perinatal lesions of the corticospinal system give rise to subtle but observable changes in spontaneous general movements without giving rise to the traditional neurological signs observed in older children and adults (9, 10).

### Periventricular white matter injury

Periventricular white matter injury is commonly seen in premature and low birth weight babies. It leads to lesions which range from regions of hypomyelination up to cystic lesions of the sub-cortical white matter adjacent to the external angles of the lateral ventricles (12) that largely leave the cortical gray matter intact, although cortical projection neurons may subsequently make aberrant intracortical axonal projections (13) and neuroimaging and neuropathological studies do show some reduction of cerebral cortical gray matter volume and reduced gyrification (14–16). PVWMI is the most important cause of cerebral palsy in prematurity and its incidence, along with the severity of cerebral palsy, have actually increased over time as medical advances have led to a greater survival rate for premature infants (17). Its etiology is multifactorial and possibly combinatorial, involving both prenatal and perinatal factors that may include genetic causes, ischemic-reperfusion failure, growth factor deficiency, and infection or inflammation ante- or postnatally (18, 19).

Thus age dependent regional susceptibility is a major characteristic of PVWMI with the highest susceptibility in the human brain between 24 and 32 weeks post-conceptional age (PCW); a stage of vascular development that leaves the periventricular regions at risk of hypoperfusion and hypoxia (20). Lesions occurring in PVWMI are located at the termination of major cerebral vessels in a border zone between anterior and middle and posterior cerebral arteries (21). These termination areas or “watershed areas” are located most distal from direct blood supply and are poorly vascularized (22). The temporal window during which PVWMI occurs closes between 30–32 weeks PCW, coincident with a marked increase in vascular supply to the white matter (23).

**Figure 2:**
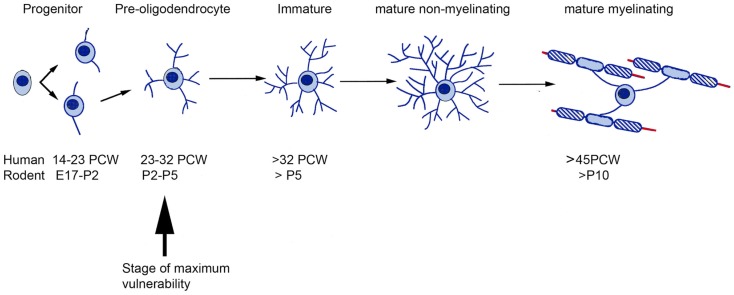
**A comparative timetable of oligodendrocyte development between rodent and human**. The time of greatest vulnerability to hypoxia/ischemia (arrow) is at the pre-oligodendrocyte stage of development. Based on the information from Ref. (34–36). E, Embryonic day; PCW, post-conceptional weeks.

At these vulnerable stages of development, the white matter grows rapidly. This requires more energy but at the same time distance from the blood vessels is increased. The combination of these factors explains why the white matter is particularly vulnerable to asphyxia, hypoxia, ischemia, and trauma (13). The sub-cortical white matter is populated predominantly by premyelinating oligodendrocytes (24, 25) including precursor cells and immature oligodendrocytes. Such cells are more vulnerable than mature oligodendrocytes to a variety of H/I injury-related insults including glutamate receptor-mediated excitotoxicity (26, 27) and glutamate transporter malfunction (28, 29) as well as arrested development (30, 31), which may arise out of oxidative stress on the cells (32) or inhibition of differentiation by extracellular components of any astrocytic scar (33). A comparison between the timetables for oligodendrocyte production, maturation, and myelination in human and rodent forebrain is presented in Figure 2.

Developing white matter is vulnerable to intra-uterine infection. This can cause severely altered fetal pulmonary function and cardiovascular control, contributing to H/I brain injury, while pro-inflammatory cytokines can interact directly with various cell populations in the brain (19, 37). In particular, the external angles of the lateral ventricles, a “crossroads” site for various axonal projections, are a location for accumulation of microglia cells, which may be involved in axonal guidance but also provide a substrate for an enhanced inflammatory reaction in PVWMI (38) producing pro-inflammatory cytokines, as well as excitotoxic glutamate and free radicals (32, 39, 40). Pro-inflammatory cytokines are also able to disrupt glutamate homeostasis and inhibit glutamate transport in oligodendrocytes and astrocytes (29, 41).

In addition to white matter injury, the transient subplate zone of the developing human cortex peaks in size between 24 and 32 PCW (42). It is located between the periventricular white matter and the smaller, developing cortical plate and has been shown to be vulnerable to H/I injury in the preterm (43). It is relatively more mature than the cortical plate, having a better developed synaptic circuitry (44) and a higher expression of glutamate receptors making its neurons relatively more vulnerable to excitotoxic injury (45, 46). Subplate neurons play an essential role in the development of connections between thalamus and cortex and of connections within the cortex (47, 48). The time period of vulnerability to PVWMI, with its secondary damage to axon tracts and to subplate neurons, coincides with the timing of thalamocortical and cortico-cortical (49) and corticospinal synaptogenesis (50) and thus can be viewed as perturbing the trajectory of sensorimotor development at a crucial stage leading to aberrant development of connectivity and mapping of functions (51, 52).

### Perinatal stroke leading to spastic hemiplegia

The incidence of stroke is highest in prematurely born babies compared to any other time of life and is also high for babies born at term (53). Two-thirds of children who suffer from perinatal stroke develop cerebral palsy and nine tenths of these will develop hemiplegic cerebral palsy (54). The outcome after adult onset stroke is largely determined by the extent of the initial brain injury and motor recovery occurs if a critical amount of corticospinal system function has been spared (55). However, this is not the case for a perinatal stroke and infants with a significant corticospinal projection from the infarcted cortex soon after the stroke, detected by transcranial magnetic stimulation (TMS), can still have a poor motor outcome (56). A longitudinal study has shown that in the first 24 months after stroke, progressive loss of corticospinal projections from the affected cortex may occur. Findings at 24 months were predictive of outcome; those in whom TMS failed to evoke responses in the affected limb had a poor outcome, failing to develop functional use of their paretic hand, whilst those in whom a response has been preserved had a better outcome, developing functionally useful dexterity in childhood (56).

After a unilateral stroke, although a corticospinal projection may be present, activity in the infarcted cortex is suppressed. Thus it has been proposed that surviving, but not very active, corticospinal projections may lose out in competition for spinal cord synaptic space, leading to these projections being withdrawn as their potential targets are taken over by more active ipsilateral corticospinal projections from the unaffected hemisphere and also by proprioceptive muscle afferents (51, 57).

## Comparisons between Species

### Periventricular zones and subplate

As discussed above, hypoxic-ischemic lesions in very premature babies target the proliferative zones around the lateral ventricles, the developing white matter tracts and subplate. At what stage of development are these structures comparable to human in our animal models? In rodent, ages ranging from embryonic day (E) 18 to post-natal day (P) 7 as the time of insult have been proposed to model human lesions in the early third trimester.

White matter vulnerability is developmental regulated, and it has been related to the presence of pre-oligodendrocytes in developing axon tracts of the forebrain during the time of peak incidence of PVWMI (see Section “Periventricular White Matter Injury”, Figure 2). In the neonatal rat, pre-oligodendrocytes are predominant in the corpus callosum and cortex between P2 and P5, whereas, immature oligodendrocytes predominate by P7 (58). Both *in vitro* and *in vivo* experiments have provided the evidence that the pre-oligodendrocytes are much more susceptible than immature oligodendrocytes to oxidative stress (59), oxygen–glucose deprivation (27), and glutamate receptor-mediated excitotoxicity (26, 60, 61). Transient synapses between growing axons and pre-oligodendrocytes play an important role in white matter development (62–64) and these are rapidly lost during hypoxic-ischemic episodes, prior to any cellular loss (65). Diffuse hypomyelination was seen in response to injections of excitotoxic ibotenic acid (IBA) into the periventricular white matter at P5 but not at P7 (66).

Therefore, most experimenters model PVWMI in rodents by delivering an H/I or excitotoxic lesion (67, 68) during the period P2–P5. At this very early stage, the corticospinal tract (CST) has reached the spinal cord but has barely begun innervating the gray matter (69). Thalamic afferents are making global, rather than lamina specific, connections throughout the cortical plate and subplate (70). Spontaneous movements, generated by bursts of activity in the spinal cord, feedback sensory information to the somatosensory cortex producing gamma oscillations followed by spindle shaped bursts of oscillatory activity (71, 72). Similar processes are occurring in human development between 24 and 32 post-conception although cortical oscillatory bursts may continue until birth (73, 74). This synchronized oscillatory network activity is proposed to drive the generation of cortical circuits (75). Thus it would appear that the period white matter vulnerability in rodents and humans is broadly comparable in terms of the stage of development of corticospinal and thalamocortical connectivity, arguably making rodents an appropriate model at this age.

The other major target for periventricular injury is the subplate, which is strikingly different in humans and rodents. In any species, the subplate is a highly dynamic compartment containing both stationary and migrating glutamatergic and GABAergic neurons, various corticopetal and corticofugal projections, glial cells, and blood vessels (48, 76, 77). In rodents, most of the subplate cells are in a thin band separating the white matter from layer 6, but some scattered cells in the upper intermediate zone are also considered to be part of the rodent subplate (78). In primates, the proportion of the subplate in relation to the rest of the cortical compartments is much greater (79). In human, the subplate zone proper becomes visible as a cell-poor/fiber-rich layer situated between the intermediate zone and cortical plate (79, 80) at around 14/15 PCW. It forms from the merging of the deepest layer of the cortical plate, with an already formed pre-subplate that contains few neurons but a differentiated neuropil featuring dendritic arborizations (81) and synapses (79), which include GABAergic elements (82) and monoaminergic innervation from the brainstem (83). This coincides with the invasion of the subplate region by thalamocortical afferents and basal forebrain afferents (84–86) as causing rapid expansion of the subplate so that it comprises a third of the cerebral wall by 16 PCW.

Birth-dating studies in rodent reveal that the subplate is among the earliest generated and earliest maturing cortical neuron population (87, 88) and in rat, becomes distinct structure from around embryonic day E16–18 (89). In contrast, in primates, neurons are continuously added to the subplate until relatively late stages of corticogenesis, including glutamatergic neurons (80, 90, 91). The subplate reaches its maximum thickness at the late second and early third trimester, and thereafter the subplate gradually decreases in size and becomes unrecognizable around the sixth post-natal month (79). The beginning of subplate neurogenesis and the arrival of the first GABAergic neurons in the subplate occur at similar stages in rodent and human (92). However, the continued addition of neurons to the primate subplate and the relatively larger proportion of the cortical wall it occupies represent major differences at later stages. Furthermore, the human subplate is compartmentalized, with neurons of different phenotypes (82, 92) and different axonal pathways (15, 77, 93) appearing in deep and superficial layers.

In summary, any lesion to the developing cortex is likely to occur at a time point when the subplate is very different in rodent and human. The human subplate will contain more glutamatergic neurons, perhaps giving greater scope for excitotoxic damage. The role of the subplate as a waiting zone for the massively increased number of intracortical fibers seen in primates will not be explored in rodent models. For instance, a recent study that explored the effect of *in utero* hypoxia at E18 on the subplate and subsequent cortical development in rodent (94) targeted the early subplate when human and rodent are more similar, but would be a model of a lesion caused during extreme prematurity in human and thus of limited clinical relevance, although otherwise of great interest from a developmental neuroscientist’s perspective.

### Corticospinal system

A major factor in the development of spastic cerebral palsy is injury to the sensorimotor cortex and its sub-cortical white matter. Our ability to model cerebral palsy is crucially dependent on understanding similarities and differences in the corticospinal system function and development in human and other species. Corticospinal projections act in parallel with a number of other descending pathways and their fields of termination overlap. In addition, the sensorimotor cortex, as well as making direct connections to the spinal cord, also connects with the origins of the other descending pathways (95). The CST provides excitation/inhibition of motoneurons, along with descending control of selection, gating, and gain control of exteroreceptive and proprioceptive sensory afferent inputs, as well as mediating plasticity in spinal cord circuits (95, 96). All descending pathways function as part of a large network rather than as separate controllers of spinal cord centers, and the spinal cord, along with segmental inputs, are part of the network.

**Figure 3:**
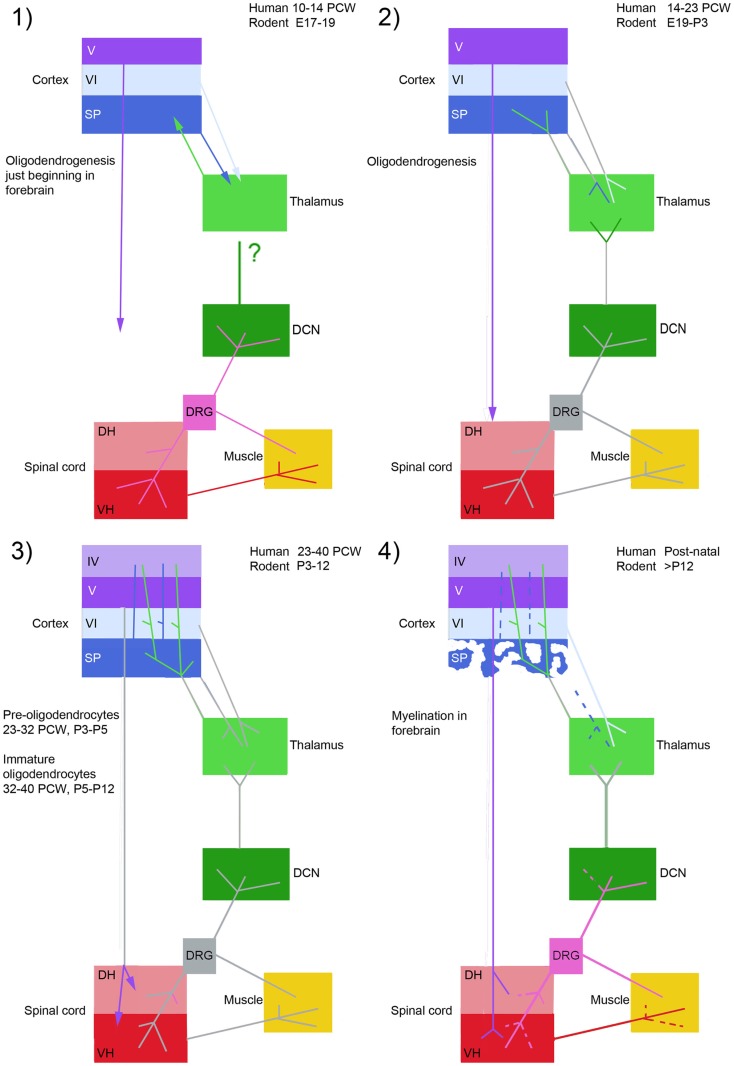
**This figure compares four stages of development of the corticospinal system in rodent and human**. At stage 1 segmental circuits are connected, and local circuitry is also forming in the forebrain, but there is no connectivity between the two. Stage 2; thalamic afferents invade the subplate, and the corticospinal tract waits in the white matter to innervate the spinal cord gray matter. Stage 3; thalamic afferents innervate layer IV of the cortex at the same time as corticospinal fibers innervate the spinal cord, thus the spinal cord and sensorimotor cortex become reciprocally connected. Spindle bursts in response to spontaneous movement are recorded in somatosensory cortex. Stage 4; the subplate dissolves and corticospinal connections and muscle afferent projections are refined in the spinal cord and dorsal column nuclei. DCN, dorsal column nuclei; DH, dorsal horn; DRG, dorsal root ganglion; SP, subplate; VH, ventral horn; IV, V, VI, cortical layers. Arrows represent ingrowth of axons, dashed lines withdrawal of axon terminals. Axon projections colored gray have not changed at that stage in the figure. Based on information from Ref. (42, 50, 57, 75, 92, 100, 101).

Developmental damage to the cortical component not only removes this element of motor control, but, as has been already been alluded to (see Section “Perinatal Stroke Leading to Spastic Hemiplegia”), removes an important influence on the way in which this distributed network is developing. Although it has often been proposed that the developing motor system has increased plasticity with which to compensate for these deficits (97, 98) there is also abundant evidence that aberrant plasticity leads to the increased and different symptoms seen in cerebral palsy compared to adult stroke (51, 99). Therefore, in choosing an animal model and interpreting the results of lesions we need to know how, and the extent to which, the sensorimotor cortex plays a role in the motor control network, and how it develops. A comparative timetable of development between rodent and human is shown in Figure 3. As the figure shows, to begin with, spinal cord and sensorimotor cortex develops independently, but at the same time as corticospinal axons begin innervate the spinal cord gray matter, ascending thalamic afferents begin to innervate layer IV of the somatosensory cortex. At this stage, damage to one element of the system, CST or subplate, perturbs development of the whole system.

Rodents have a CST that projects the full length of the spinal cord (102, 103) and is involved in fine movement control (104) however, the primate CST arises from a proportionally larger area of the cerebral cortex (105), possesses a fast-conducting component and the corticospinal axons are largely situated in the lateral, not dorsal, columns of the spinal cord, as they are in rodents. Kittens have also been studied as they also have the advantage of being born early in the development of the motor system, and that there is a wealth of previous research on the feline locomotor system (96).

Differences between rodents and primates in the pattern of CST terminations are both qualitative and quantitative. In rodents, the CST almost entirely projects to dorsal horn neurons and premotor spinal circuits (102, 103). In many non-human primates, such as the rhesus monkey, the projection pattern of the CST is much more complex: a significant proportion of CST fibers projects to the ventral horn, and some axons synapse directly on motoneurons, in particular those innervating hand muscles (106). In humans, this trend is even more marked (107). For example, there is a strong correlation between the number of direct connections between cortex and motor neurons and the level of manual dexterity of non-human primate species (106, 108). Rodents have very few, if any, direct connections (103, 109, 110) and this observation has been employed to explain a perceived relative lack of ability to control hand/paw musculature (111) although it has been claimed that rodents have more dexterity than is generally appreciated, which is impaired by CST or sensorimotor cortex lesions (112, 113). Similarly, damage to the CST in rhesus monkeys causes permanent deficits during stepping (114) as in humans where CST damage is severe enough to compromise independent walking (115). It has been claimed CST lesions have little effect on stepping in rodents (116) however, a more recent study have demonstrated that CST function is necessary for the avoidance of obstacles during stepping (117). In conclusion, although subtle, rodents do suffer deficits in skilled motor performance following injury to the CST, but these require subtle outcome measures to be detected.

There has also been an evolution in the role that parallel descending pathways play. In both cats and rodents, there is a prominent contralateral rubrospinal projection mostly from large neurons in the red nucleus to premotor neurons and motoneurons in the spinal cord (118–121). This is greatly reduced in macaques, which have, instead, an expanded rubro-olivary projection from small cells in the nucleus with projections from the sensorimotor cortex predominantly target the small cells (122). In human, the rubrospinal tract is greatly reduced, although still present (123, 124). Similarly, cats possess C3–C4 propriospinal interneurons that are the relays for a significant di-synaptic pathway between the cortex and motoneurons of the lower cervical cord (125) but here is no evidence for such a pathway in macaques unless inhibition in the spinal cord is greatly reduced (126). Indirect measurements have provided evidence for this pathway in humans and it may be up-regulated in patients with hemiplegia after stroke (127). However, this pathway appears not to exist in rodents (110). Cortico-reticular pathways to the spinal cord, including direct projections to motoneurons in monkeys (128, 129) have been described, although it is worth noting that even in rodents there are inter species differences with mice having a much weaker excitatory pathway than rats (110). Exactly what plasticity may occur in unlesioned descending pathways is species dependent, and this needs to be taken into account when interpreting animal models.

Another important consideration is the extent of the ipsilateral CST. In macaques it is quite large; 13% of all corticospinal axons fail to decussate in the medulla (130) and this is similar to the human CST (131) whereas in rodents only 2–4% remain uncrossed (132). The adult ipsilateral projection is also similarly small in the cat (133). To confuse matters more, in monkeys there are bilateral projections and fibers crossing from the ipsilateral to contralateral side at the spinal cord segmental level, as well as contralateral axons re-crossing to terminate on the ipsilateral side (130, 134) but in rodents nearly all corticospinal axon terminate without crossing the spinal cord midline (135, 136). However, it should be born in mind that ipsilateral corticospinal connections in the monkey cervical spinal cord are different from contralateral projections as they fail to make monosynaptic connections with motoneurons (137).

Plasticity in the ipsilateral tract following a perinatal hemiplegic stroke could provide a gateway to improving function in the affected limbs. Surprisingly, there is evidence for an extensive transient ipsilateral projection in humans, where, in the newborn, TMS is as likely to produce ipsilateral contractions in arm muscles as it is contralateral muscles, only with a shorter latency, suggesting a direct projection (56). These ipsilateral projections are down-regulated during normal post-natal development, however in patients with hemiplegia derived from a pre- or perinatal lesion, or developmental malformation, these ipsilateral connections are retained (56, 138, 139), although they confer no functional advantage (56). Hypertrophy of the pyramid contralateral to the lesion has been interpreted as showing that the fibers are retained projections from neurons in the intact hemisphere normally lost during development (56). To what extent this can be modeled in animals is discussed in Section “Modelling Corticospinal Plasticity.”

Fast onset, low threshold, and aberrant reflex pathways are observed in spastic cerebral palsy sufferers (140, 141) may result from retention of developmental reflex pathways in the absence of corticospinal input at a crucial stage of development (57). In human and rodents alike the excitation threshold of stretch reflexes increases with age (142, 143). This may partly be because muscle afferents first target the cell bodies and proximal dendrites of motoneurons in both rodent and human (144–147) although, in maturity, these afferents principally target more distal dendritic sites (148).

Activation of the stretch reflex in the biceps brachii of a newborn human also results in fast heteronymous excitation of antagonist muscles such as triceps brachii, providing evidence for the existence of superfluous connectivity that is presumably eliminated later in development (143). However, in rodents much research suggests that muscle afferents innervate homonymous and synergistic motoneurons with a high degree of accuracy from the outset (149–151). Nevertheless, patterns of muscle afferent innervation change with development in the rodent ventral horn (146) and cuneate nucleus (101) and in the intermediate gray (152) of the kitten spinal cord. Therefore, it may be possible to study some aspects of aberrant spinal cord development in response to cortical lesion, but the high degree of spasticity and aberrant reflex formation observed in humans is not be substantially reproduced in rodents.

## Critical Examination of Animal Models in Use

### Models of periventricular white matter injury

Based on the various risk factors discussed in Section “Cerebral Palsy in Humans,” various animal models have been developed in different species but mostly rodents, including models of hypoperfusion and models using infectious agents, bacterial products, or excitotoxic insults. These varied approaches were extensively reviewed by Hagberg et al. (67) and their recommendations have strongly influenced the field ever since. Approaches used in rodents fall into two main classes; firstly, the induction of H/I by the maintenance in a hypoxic environment for a period of time, coupled with unilateral ligation or cauterization of the common carotid artery, the Rice–Vannucci model, which has been use for over 30 years and has the advantage of being extremely well characterized (153). The drawback is that although the lesion is reproducible and bears some resemblance to lesions observed in affected infants, the method for inducing it is artificial. Also, this approach is generally employed at P7 or slightly later, and as discussed in Section “Cerebral Palsy in Humans,” the period of peak oligodendrocyte vulnerability occurs a little earlier (Figure 2). Thus, although the Rice–Vannucci method recently has been applied at earlier ages [e.g., Ref. (31, 154)] because of the difficulty of employing the Rice–Vannucci approach at younger ages other approaches involving modeling the consequences of hypoxia have also been employed including intracerebral injection of excitotoxic agents (66, 68, 155, 156) or agents causing oxidative stress (157). Hypoxia on its own has also been employed, for instance gestational hypoxia between E5 and E20 in rats induced white matter damage due to a local inflammatory response and oxidative stress linked to re-oxygenation during the perinatal period (158) however, the relevance of this model to most cases of cerebral palsy is not clear.

Systemic or intracerebral injection of inflammatory agents between P3 and P7 has also been employed (159–161). These approaches again yield reproducible lesions but only model some aspects of the human condition. Because intra-uterine inflammation may be a significant contributing factor to brain injury leading to cerebral palsy, many animal models have been developed in which intra-uterine inflammation is instigated in rodents and rabbits prior to birth [reviewed by Burd et al. (162)]. The significant drawback with these experiments is that they are instigated very early in development, as the species are born at a very premature stage of development compared to humans (see Figure 2). For instance, some experiments have taken place at E9–10 in mouse (163–165) at a time when neocorticogenesis is only just beginning [7–8 PCW in human (166)] and this really only suitable for modeling proposed neurogenesis and cell migration deficits seen in neurodevelopmental disorders such as autism or schizophrenia. Even studies toward the end of rodent gestation (167–169) or rabbit (170, 171) are modeling extreme prematurity, that is, halfway through the second trimester (166) and therefore of limited relevance to most cases of cerebral palsy.

The purpose of developing these models has included both testing early interventions for preventing or reducing PVWMI, and discovering other factors that exacerbate the condition. For instance, a model of PVWMI induced by intracerebral excitotoxin injection at P5 has been shown to be exacerbated by additional systemically administered pro-inflammatory cytokines and interleukin-9 (172) helping to establish the multifactorial nature of the condition. Similarly, excitotoxic lesions were significantly worsened in mouse pups exposed to gestational stress caused by a significant rise of circulating corticosterone levels both in pregnant mothers and in newborn pups, acting through glucocorticoid receptors (173). Using transgenic technology, the widely expressed kinase GRK2 has been implicated in protecting white matter against H/I injury (174) suggesting that genetic variability between individuals may contribute to the severity of perinatal brain damage. A recent study has revealed a novel, gender-specific protective role for innate immune receptor signaling in a mouse model of neonatal hypoxic-ischemic brain injury (175) revealing another potential source of variability in injury severity.

Testing protective interventions has been carried out in many and varied studies. For instance, the extent of injury has been reduced by administration of glutamate antagonists (26) including successful magnesium sulfate as a blocker of NMDA receptor channels (155, 176) leading to clinical trials of this approach, although not, as yet, with any convincing evidence of beneficial effects (177). A variety of other agents have been trialed pre-clinically with some promise of efficacy, including vasoactive intestinal polypeptide and melatonin, which act by modulating second messenger systems (178, 179). Stem cell therapies have been tested pre-clinically, which may modulate the inflammatory response and/or stimulate host production of new oligodendrocytes (180–185).

The antibiotic minocycline, which also inhibits the activity of microglia, has been extensively tested and reduces white matter damage and brain lesion size [e.g., Ref. (186–188)]. However, minocycline studies also provide a lesson in the problems of scaling up pre-clinical trials in rodents to human as explained by Buller et al. (189). Preconditioning dosing strategies may be more beneficial, however administration post-insult has more clinical relevance, as a diagnosis of perinatal HI in the neonate is often not made until 3 days after birth. Routes of administration appropriate for babies have not undergone trials. Large single doses exacerbate injury in mice (190) but this may be strain specific. Repeated doses of the drug appear to be more effective (188) but it is difficult to predict the length of treatment required when glial cells mature in a matter of days in rodents but over months in humans, bearing in mind the detrimental effects of tetracycline antibiotics on growth of bones and teeth (191).

As well as differences in maturation time compared to the gyrencephalic human brain, rodents also have a substantially smaller proportion of sub-cortical white matter, substantial differences in cerebral blood flow and metabolism and a greater susceptibility to gray matter injury in response to white matter lesions (153, 192). Thus, the fetal sheep has been proposed as an alternative model for a number of reasons. It is possible to perform experiments and make repeated measurements *in utero*. The stage of development of the ovine fetus at 95 days post-conception shows strong similarity with the early third trimester human, both in terms of oligodendrocyte development (193) and general brain development including in terms of the completion of neurogenesis, the onset of cerebral sulcation, and the detection of the cortical component of somatosensory evoked potentials (192). Melatonin therapy has been pre-clinically tested, with success, in sheep (194). Adaptive brain shut down and neuroinflammation have also been studied in the near term ovine fetus (195, 196). However, a significant drawback is that although sheep might provide a good model of white matter damage, they provide a poor model of corticospinal function as the CST fails to project below the upper cervical level (197) and no protocols have been developed for neurobehavioral studies of sheep receiving preterm lesions.

### Models of perinatal ischemic stroke

In human neonates, perinatal arterial ischemic stroke (PIS) events occur mostly in the middle cerebral artery (198, 199). Therefore, focal MCAO models reflect the vascular distribution seen in human neonates with ischemic stroke rather than other H/I models that more accurately model PVWMI (see above). The heterogeneous nature of PIS in human leads to two types of studies. Some investigators have used permanent focal MCAO for animal models, while others apply transient occlusion that allows reperfusion for occluded vessels. The pathology of both types is similar although the injury pattern and severity of brain injury differ. A permanent occlusion results in a severe ischemic injury accompanied by necrosis, whereas transient occlusion can produce a lower injury severity, depending on the occlusion duration, accompanied by apoptosis (200, 201). There is also apoptotic like cell death during the first 24 h in permanent occlusion models (202). After introducing these types of lesion to rat pups, two zones of ischemic injury occur; a central, necrotic injury zone with little scope for recovery, and a penumbra where apoptotic cell death is more usually seen and there is some scope for rescuing the tissue (200, 201, 203, 204).

Studies that used transient MCAO (200, 205–207) claim that their model reflects neonatal PIS since reperfusion mimics what happens to neonates when circulation is permitted by collateral circulation to the penumbral part of the ischemic lesion (208). On the other hand, studies not involving reperfusion in their MCAO model argue that there is no consistency in reperfusion among patients (209). The Left middle cerebral artery is most commonly occluded in neonatal ischemic stroke (198, 210) and so is most commonly targeted in animal models. The internal carotid artery is catheterized by monofilament suture to occlude the middle cerebral artery permanently by retaining the filament, or temporarily by removing it at the desired time (211).

This approach was first applied to young rats (P 14–18) by Ashwal et al. (205) to cause transient occlusion at the proximal middle cerebral artery followed by reperfusion. Cytotoxic edema occurred in the ischemic region immediately after the occlusion, then severe injury in a similar region occurred after reperfusion (200). A study that used high-field MRI over a 28-day period post-lesion demonstrated that transient filament MCAO models induce infarction with maximum volume at day 1–3 post-occlusion (207). Three hours of occlusion resulted in infarcts that included the striatum and affected 40–50% of the whole hemisphere and may resemble human stroke (205). However, this method produced unacceptably high mortality rates where only 21% of pups survived for more than 28 days (207). Animal welfare concerns apart, this does not allow for long term assessment of treatment outcomes. Interestingly, transient occlusion of the common carotid artery for 60–90 min, combined with permanent ligation of the middle cerebral artery produced only neocortical injury (203) however, whether occlusion of arteries external to the cranium really models human strokes is questionable. Nevertheless, such models have a lower mortality rate and can cause sensorimotor and cognitive impairments in early adulthood such as postural asymmetry, motor incoordination, and cognitive impairments, although the lesion site is small by this age (204).

The introduction of an embolus into the MCA, guided from the CCA or ECA with a filament, was pioneered by Derugin et al. (206) and further refined by individualizing embolus size to the rat’s size (202). It was claimed that the infarction pattern in their model mimics that of the MRI pattern for the human neonate (212). Infarcts in this model are located in the cortex and the striatum, and the infarcted area in the cortex is 51–56% of the ipsilateral hemisphere in the forebrain and no mortality during this time period (202).

Another approach is to ligate or electro-coagulate the distal middle cerebral artery, approached following a craniotomy, to produce permanent occlusion. MCA ligation performed at the level of inferior cerebral vein in mature and immature rats fails to cause an infarction in all animals (203, 213, 214). If applied at the level of the olfactory tract, infarction resulted in 13% of rats; occlusion at the MCA origin caused infarction in 67% of rats. To achieve 100% of rats with cerebral injury, ligation 3–6 mm along the MCA starting from its origin or proximal to the olfactory tract to the level of inferior cerebral vein is required, which would include all supplying arteries from the proximal to distal portion of MCA (214). Recently, this model was applied in neonatal Cb-17 mice producing selective and consistent cortical injury, mild corpus callosum atrophy, and mild thalamic injury similar to what is seen in infant stroke and leading to significant sensorimotor defects (209). The method is highly reproducible in this mouse strain; the operation requires <15 min and a 100% survival rate is reported. Reproducibility may be due to the small variation in cerebrovascular structure observed in these mice (215) and it advised that rodent strains with a robust collateral blood supply to sensorimotor areas, for instance Wistar rats, are avoided when contemplating these experiments (208, 216). Strain can also strongly influence the ischemic injury pattern, for instance, CD1 mice after carotid ligation on P12, are more vulnerable to epilepsy than C57Bl/6 mice, as are the C3Heb/FeJ strain (217, 218).

Alternatively, thrombosis can be induced by injecting the vascular system with a photosensitive dye and exposing blood vessels to light resulting in permanent focal ischemia (219). Permanent occlusion was produced in piglets by exposing the MCA. Severe reduction in cerebral blood flow and gray and white matter injury with 7.1–12.3% infarction volume of ipsilateral hemisphere occurred in this model (220). This has also been applied in 7-day old-rats causing direct injury to the sensorimotor cortex (221). As laser exposure duration increased, so did severity and size of the injury and the deficit in motor performance (221). Thus, infarction volume can be controlled according to the exposure time. In addition it is a non-invasive method with low mortality rate (220, 221). However, the pathogenesis of this focal ischemic infarction is of debatable relevance to human neonatal stroke.

The events of perinatal ischemia are suggested to occur any time over a period of 20 weeks that spans late fetal and early neonatal life (222). Thus human perinatal stroke are classified according to the infant age when diagnosis is made as well as radiological assessment patterns of injury (199, 222). However, the first week of life is the main period when PIS will occur (199). The use of animal models, mainly rodent, to reflect ischemic stroke in the perinatal human period depends on matching the appropriate age between human neonate and animal models by correlating neuronal events that occur during maturation. Correlating human full term to model post-natal age (P) is an area of conflict in the literature. Based on different criteria, authors claimed that human term corresponds to either P7 (223) or P8–14 of rodent age [white matter development (67); Corticospinal system development (57); and EEG maturation (224)]. Several of the earlier studies discussed above have used P7 rodents (200, 202–204, 206, 221) based on Hagberg et al. (223). Other studies have used a more appropriate age either because of the difficulty of performing experiments in younger animals (205, 207) or following Hagberg et al. (67) for example Tsuji et al. (209).

Finally, it should be born in mind that an infarction that destroys the sensorimotor cortex may not be required to model cerebral palsy. Eyre et al. (56) demonstrated that in human developmental hemiplegia during the earliest stages, a corticospinal projection is still present, which fails to develop and is withdrawn. Therefore, the aim may be to induce a degree of hypoxia that delays maturation of the cortical tissue rather than destroys it completely, and it may be that more detailed measures of outcomes are required in our animal models than the presence or absence of tissue.

### Measuring outcomes with models of PVWMI and PIS

**Figure 4:**
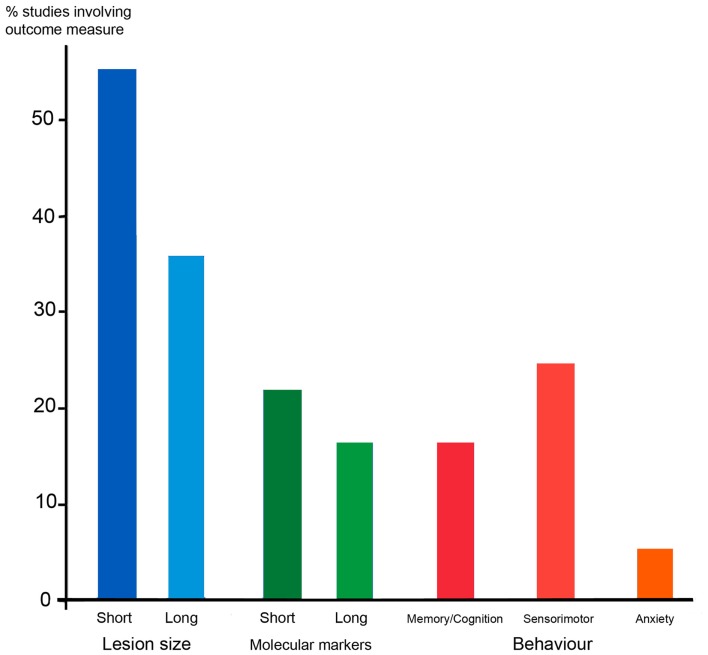
**The outcome measures employed in a sample of 36 rodent studies that modeled PVWMI or PIS, some of which involved experimental therapies**. Blue columns depict the proportion of studies that studied lesion size in the short term (within a week) or in the longer term, either using MRI, or histology. Green shows studies of changes in molecular markers in response to lesions, e.g., markers of apoptosis, myelin, and gliosis. Red/orange shows behavioral testing in adolescent or adult animals following perinatal lesions. These are divided into tests of memory and cognition (e.g., mazes) sensorimotor (e.g., rotarod, reaching, and ladder walking) and anxiety (open field). The 36 studies sampled are those involving rodents cited in Sections “Models of Periventricular White Matter Injury” and “Models of Perinatal Ischemic Stroke.”

A problem with interpreting all animal models of PVWM and PIS is the diversity of outcomes measured. We have surveyed a sample of studies in rodents, taking as our sample the 36 studies cited in Sections “Models of Periventricular White Matter Injury” and “Models of Perinatal Ischemic Stroke” above. The results are summarized in Figure 4. The majority of studies (56%) measured the lesion size within a week of the insult, but only 36% measured the lesion size in the longer term, either by MRI or histology. Less than a quarter of studies investigated changes in molecular markers, such as markers of apoptosis, gliosis, or myelination, in either the short or long term. Behavioral testing was even rarer. Testing of sensorimotor function was most common, being carried out in a quarter of studies, but cognition or anxiety has also been measured in around 17 or 6% of studies, respectively. Tests for sensorimotor function employed are not necessarily very specific tests for corticospinal function, often consisting of observing the righting reflex or rotarod performance, and rarely testing limb placement or reaching skill.

Thus it appears for most researchers, the aim is to show a reduction in brain damage inflicted by whatever lesion is employed, sometimes simply by measuring the size of the damaged area, or sometimes the extent of cell death or demyelination, often just in the short term. Of course, any treatments that can be proven to ameliorate the effects of H/I, if given early, are of value. Also, cerebral palsy is not the only, or even the most common, outcome of early brain injury and it is important to access the effects on brain function other than sensorimotor co-ordination. But as has been discussed above, and will be further explored below, the mal-development of the sensorimotor system following a lesion is protracted and complicated, and animal experiments designed to model cerebral palsy must try and find ways of addressing this problem. It is paradoxical that, in human, we have long been adept at recognizing and quantifying the neurological symptoms of cerebral palsy, and only more recently have been attempting to measure the more subtle signs of deficits in cognition and attention. In animal models, it has so far been easier to measure lesion size, or standard behavioral tests such rotarod, water maze, and open field. Evidence of corticospinal deficits is harder to observe and test for, and this is the topic of the next section.

### Modeling corticospinal plasticity

Spastic cerebral palsy is primarily a lesion of the CST, which results in secondary maldevelopments of related circuitry, which may include a retained ipsilateral tract and aberrant development of spinal reflex pathways (see Section “Perinatal Stroke Leading to Spastic Hemiplegia”). Might it be possible to gain useful understanding of these processes by making a controlled lesion of the sensorimotor cortex that do not necessarily mimic the injuries observed in a clinical setting? Such approaches have been adopted including aspiration of brain tissue, prolonged inhibition of areas of cortex by slow release of pharmacological agents, or genetic ablation of corticofugal tracts.

An increased ipsilateral projection has been reported following developmental unilateral lesions in animal models but the nature of the projection varies depending on the timing of the lesion and the species involved. For instance, in rodents it appears that lesions made in the first week of birth, when the majority of the corticospinal fibers are growing into the spinal cord (Figure 3) results in an enlarged ipsilateral projections that predominantly comprise a non-decussating pathway, or a double decussating pathway (132, 225–227). However lesions at P7 or later tend to cause branching of fibers to innervate both sides of the spinal cord (228, 229). There is no evidence for a transient ipsilateral CST in development that is proportionally larger than in maturity, in either developing rodents (230) or monkeys (134) although as the projection from cortex to spinal cord is generally from a larger proportion of the cortical surface in development than in maturity, there may still be a proportionate withdrawal of ipsilateral axons. On the other hand, in kittens corticospinal fibers initially branch and bilaterally innervate the spinal cord (231). Under normal circumstances, the transient ipsilateral projection is withdrawn whereas the contralateral projection expands and reinforces its synaptic connections (133, 231). However, the ipsilateral projection can be maintained by removing the competing contralateral projection (232) or blocking its activity pharmacologically by continuously infusing the gamma-aminobutyric acid (GABA)-agonist drug muscimol within the developing motor cortex (233). Neural inactivation is performed between post-natal weeks 5 and 7, a developmental period during which most transient dorsoventral and ipsilateral terminations are eliminated (233, 234).

Martin and colleagues have used their unilateral cortical inactivation model in kittens to test two therapeutic strategies. Firstly, the affected CST was electrically stimulated daily over three weeks between post-natal weeks 8 and 11 (235) secondly the previously uninvolved contralateral cortex was chronically inhibited at this time (236). Both methods restored and strengthened contralateral CST connections to their normal spinal targets in the intermediate gray matter and reduced aberrant ipsilateral connections. They also led to motor recovery in a visually guided motor task. This suggests that it is balancing activity in the two competing tracts that leads to correct distribution of corticospinal inputs, not the amount of activity *per se*. Their studies were extended to non-invasive behavioral approaches mimicking potential interventions in infants (237) involving restraint of the non-involved limb with or without reach training in kittens or young cats (238). Interestingly, all three interventions restored normal contralateral corticospinal termination patterns but did not reduce aberrant ipsilateral connectivity. Only limb restraint combined with reach training restored behavior. This showed that factors additional to restoring CST connectivity contribute to motor recovery. These include re-establishing a motor map, which was only achieved with reach training.

Although these experiments in kittens appear to give useful pointers to therapies for early interventions in hemiplegic cerebral palsy, the situation as hypothesized in humans requires the presence of a large transient *unbranched* ipsilateral projection that is retained following a unilateral lesion (56, 239). Possibly any human transient ipsilateral projection is actually quite small but is still able to excite motoneurons directly, owing to the greater excitability of immature motoneurons (240) in which case rodents receiving a lesion before post-natal day 7 (which have unbranched ipsilateral projections, see above) may provide an accurate model. Alternatively, bilateral excitation of motoneurons from motor cortex may result from up-regulation of a fast pathway via cortico-reticular synapses, as reticular neurons bilaterally innervate motor columns including those innervating hand or paw muscles (109, 241, 242). This form of plasticity has yet to be adequately explored in developmental models (243). Interestingly, following hemi-decortication in rat at P5, aberrant connections were formed from the surviving motor cortex to contralateral red nucleus, superior colliculus, pontine nuclei, and the ipsilateral dorsal column nucleus and cervical spinal cord, which preserved forelimb function, but no aberrant projection to reticulospinal neurons was seen (244) perhaps because a bilateral corticoreticular projection is already present.

Simple lesion experiments have explored the extent to which normal development of intrinsic spinal cord circuitry, which extends beyond the period of CST innervation (57) depends upon a functional CST. Unilateral lesions to the sensorimotor cortex (245) or spinal cord transection during development (246) in rodents leads to retention of muscle afferents in the ventral horn and strengthened segmental reflex pathways. This is possibly analogous to the fast onset, low threshold, and aberrant reflex pathways that are observed in spastic cerebral palsy sufferers (141). Both muscimol blockade, and lesioning of the sensorimotor cortex unilaterally at P7, when the CST begins to innervate spinal cord gray matter (Figure 3) prevented the normal up-regulation of expression of the activity dependent marker parvalbumin in spinal cord neurons contralaterally (245, 247, 248) in rat. A recent study in mouse, in which corticospinal input was removed entirely by genetic ablation of all cortifugal outputs, did not result in loss of spinal cord parvalbumin expression (249). This might be explained by species differences, but it seems possible that an imbalance in activity, rather total loss of inputs, is required to cause some alterations in gene expression. Changes were seen in other interneuron subgroups and in motoneurons, including increased detection of cholinergic interneurons (249).

An increase in spinal cholinergic interneurons between 4 and 8 weeks postnatally in kittens is another late developmental event coincident with the re-organization of corticospinal input (250). Inactivation of the developing CST, and resulting motor impairments, significantly reduces the number of spinal cholinergic interneurons unilaterally, again highlighting possible differences between unilateral inactivation and total genetic ablation. Constraint combined with early reach training resulted in increases in number of cholinergic interneurons on the injured side of the spinal cord, far more than constraint alone or in combination with late reach training. Thus, behavioral recovery was associated with the substantially larger cholinergic interneuron response (238). Because these spinal interneurons are excitatory, they may augment the effect of CST input to spinal cord circuitry. What is required now is evidence that cholinergic interneurons play a role in human spinal cord function and development.

## Conclusion

When considering the outcome of testing experimental therapies for cerebral palsy in animal models it is important to ask a number of questions. Firstly, what type of cerebral palsy are we modeling? As this review has shown, the timing and nature of the lesion can be varied to model different types. Secondly, are we causing behavioral deficits typical of the human condition? Rodents do not suffer spasticity or severe locomotor impairment in response to sensorimotor cortex lesions, but there is evidence of subtle, CST dependent sensorimotor deficits that can be quantified. This leads onto the third point, is re-organization of the CST the same in our models following lesion compared to human? It is clear that primate CST organization is quite different from rodents or kittens, and modeling, for instance, ipsilateral pathway plasticity is fraught with difficulty. Finally, one of the trickiest problems with rodents is the rapid development of nervous system, which can take place in the time it takes for post-lesion inflammatory processes to take place, which poses the question can we intervene quickly enough in rodent models to change the course of maldevelopments in the motor system?

It might seem that a serious pre-clinical trial of a therapy ought to include non-human primate experiments, and yet there is only one model of perinatal H/I injury in primates that has been developed, to our knowledge. This involved focused lesions to the visual cortex caused by injection of endothelin to constrict blood vessels in the P14 marmoset (251), which caused similar anatomical and cellular pathology to that observed in post-ischemic humans at a stage of visual cortex development equivalent to 3–5 months postnatally in the human. However, very little is known about sensorimotor cortex/CST development in any primate species, and so knowing when to carry out lesions would be difficult. CST ingrowth into the ventral horn and development corticomotoneuronal synapses occurs postnatally in macaque (252, 253) but by concentrating on the elaboration of corticomotoneuronal connections to hand muscles originating from specific areas of the motor cortex, these studies ignored the higher density of corticospinally projecting neurons, coming from a larger area of cortex, in the neonate compared to the adult, as detected by retrograde tracing experiments (134). Thus a whole process of corticospinal axon elaboration and refinement, including elimination of transient projections including ipsilateral axons and projections from non-motor areas, as has been proposed for human development from indirect observations (51, 254) may, or may not, be present in the non-human primate.

As always, more research is needed, but the considerable difficulties of doing even basic research on motor development in non-human primates, let alone using them for neonatal lesion studies, would seem to make it unlikely that this line of research will be frequently taken in the future, in which case it is vital we understand the limitations of translating pre-clinical research in rodent and other species to human cerebral palsy. We hope this review may be of some help in making those judgments.

## Conflict of Interest Statement

The authors declare that the research was conducted in the absence of any commercial or financial relationships that could be construed as a potential conflict of interest.
